# Supplementary material for: Arbuscular-Mycorrhizal Symbiosis in Medicago Regulated by the Transcription Factor MtbHLHm1;1 and the Ammonium Facilitator Protein MtAMF1;3
Source: Int J Mol Sci. 2023 Sep 19;24(18):14263. doi: 10.3390/ijms241814263 (PMC10532333; doi:10.3390/ijms241814263)
Supplement: Supplementary file 1 [file ijms-24-14263-s001.zip › ijms-2539762-supplementary.pdf]

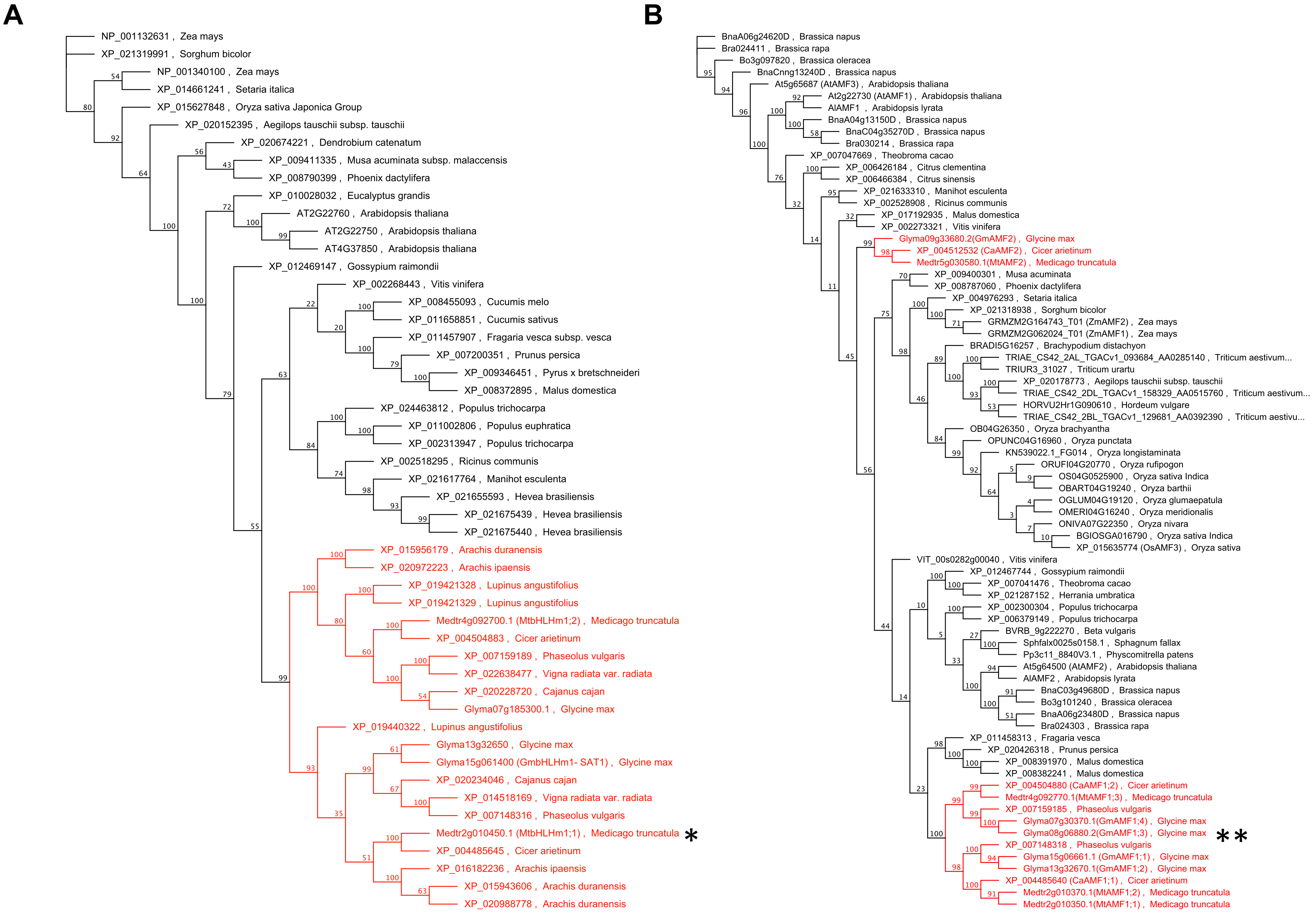

Supplementary Figure S1. Phylogeny of bHLHm1 and AMF proteins. A, bHLHm1 orthologous group: MtbHLHm1 is indicated by *; **B**, Phylogenetic tree of AMF proteins, MtAMF1;3 is indicated by **. The tree was developed using PHYML with MTREV substitution model with Bootstrap (500) branch support. The tree was drawn with equal branch lengths with bootstrap proportions indicated.

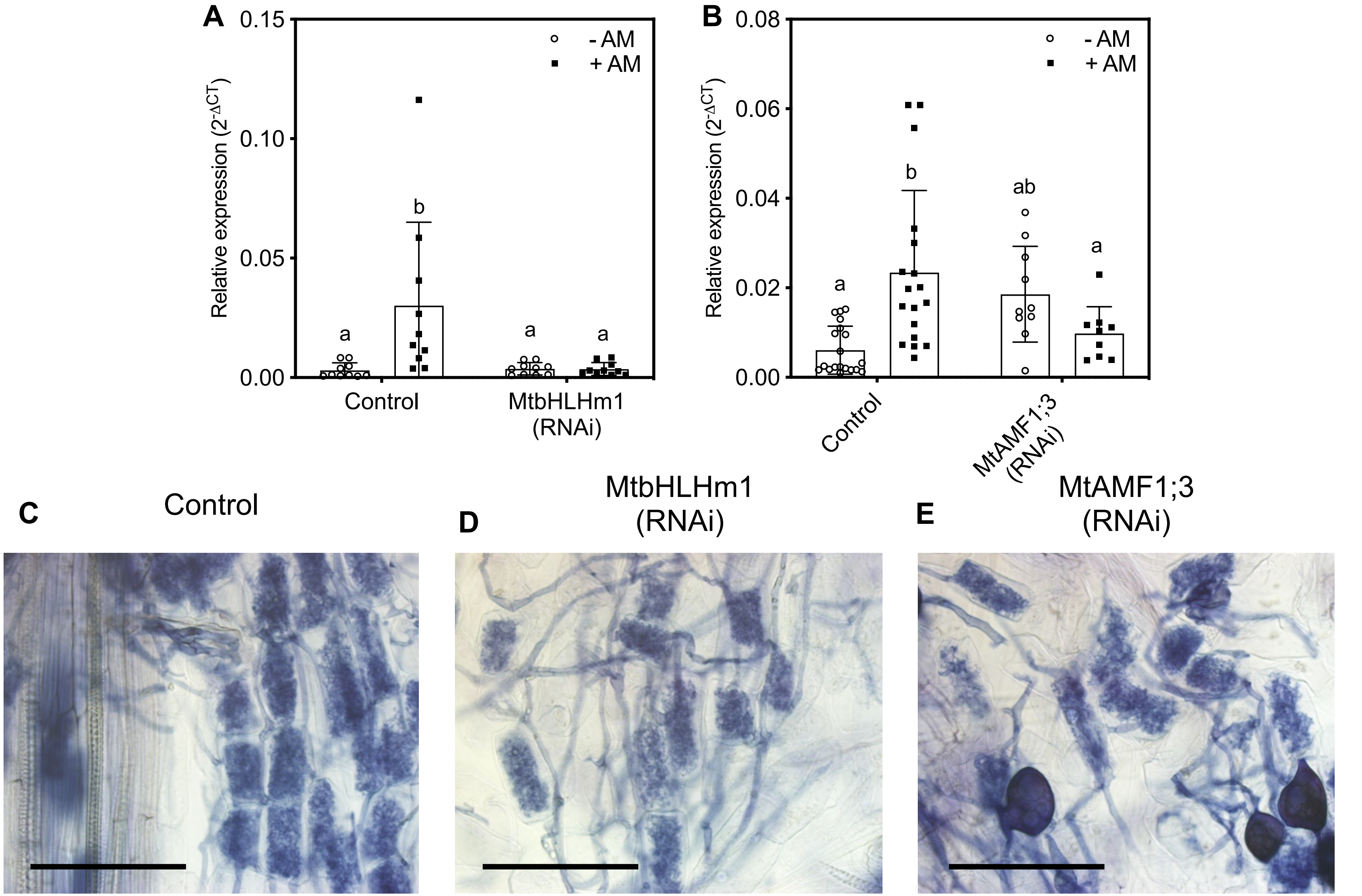

Supplementary Figure S2. RNAi silencing of MtbHLHm1 and MtAMF1;3. A and **B**, Gene expression analysis by RT-qPCR. Values indicate relative changes in expression relative to *UBQ10* ($2^{-\Delta CT}$). **A**, Empty vector control or *35S*$_{pro}$*::MtbHLHm1* (RNAi) roots colonised without or with AM fungi. **B**, Empty vector control or *35S*$_{pro}$*::MtbAMF1;3* (RNAi) roots colonised without or with AM fungi. Data in A and B represent the mean ± SD (n=10-20 individual plants). Significance was determined using two-way ANOVA with a multiple comparison test (Sidak). Different letters indicate significance between -AM and +AM colonised roots ($p<0.05$). **C-D** Overviews of ink-stained mycorrhizal hyphae and arbuscules in roots, from left to right: **C,** Empty vector (Control), **D**, *35S*$_{pro}$*::MtbHlHm1* (RNAi) and **E,** *35S*$_{pro}$*::MtAMF1;3* (RNAi) Scale bar: 50 µm

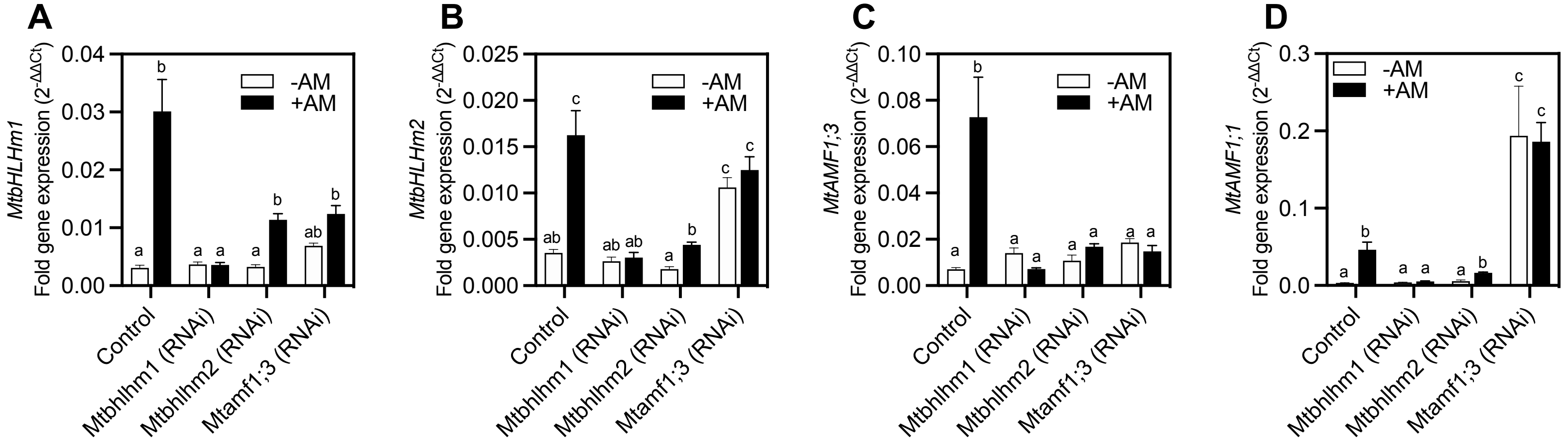

Supplementary Figure S3. RNAi silencing of *Mtbhlhm1*, *Mtbhlhm2* and *Mtamf1;3*. Gene expression analysis by RT-qPCR. Values indicate fold-change in expression relative to *UBQ10* [48] without (-AM) or with (+AM) mycorrhizal inoculation. **A**, *MtbHLHm1* expression, B, *MtbHLHm2* expression, C, *MtAMF1;3* expression and D, *MtAMF1;1* expression in roots of empty vector (Control), *35S*$_{pro}$*::MtbHLHm1* (RNAi), *35S*$_{pro}$*::MtbHLHm2* (RNAi) or *35S*$_{pro}$*::Mtamf1;3* (RNAi) roots. Data represents the mean ± SD (n=5 individual plants). Significance was determined using a two-way ANOVA with a multiple comparison test (Sidak). * indicates significance at $p<0.05$.

.

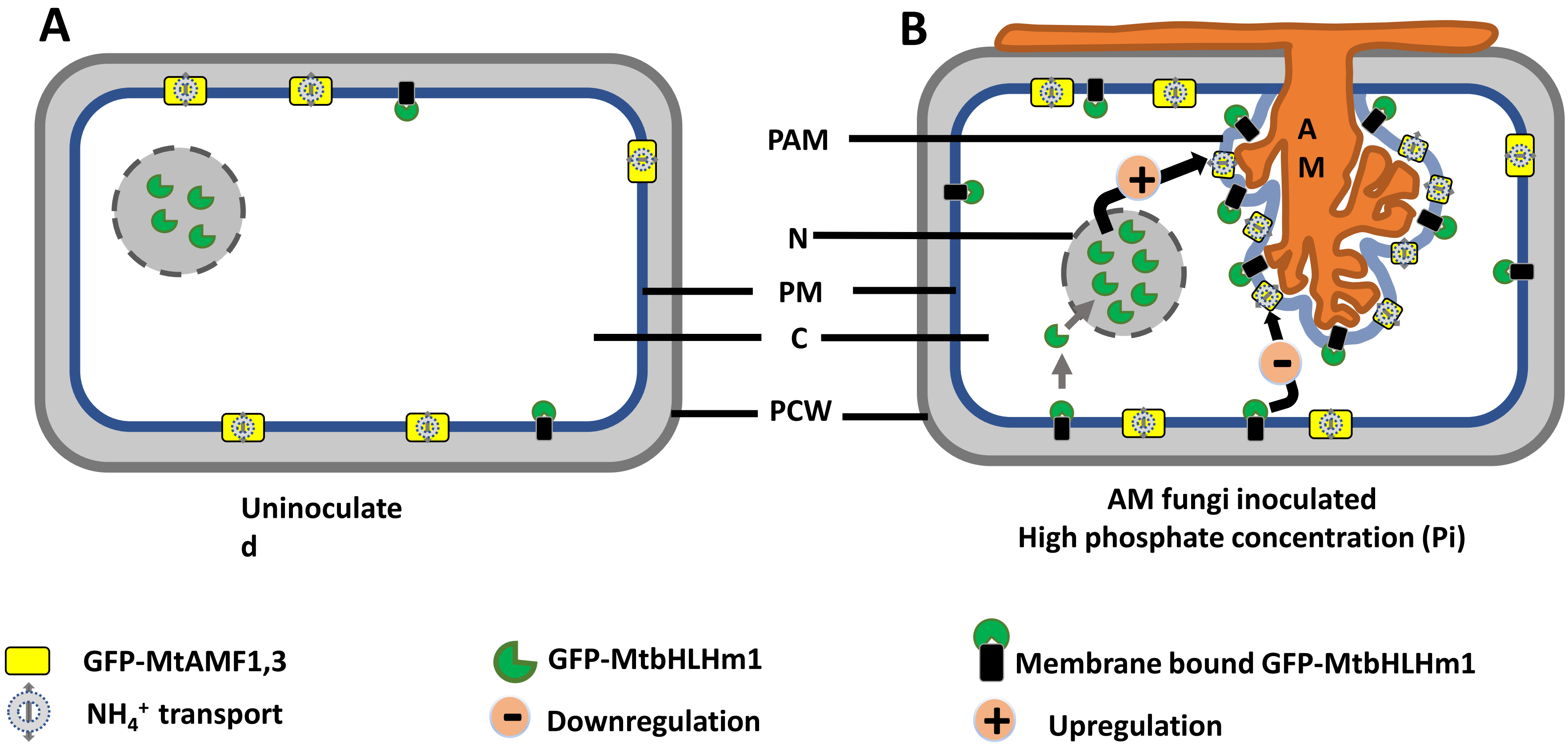

Supplementary Figure S4: Schematic model of the AMF1 regulation by the rhizobial transcription factor bHLHm1 within the inner cortical cells of *Medicago truncatula* during arbuscular-mycorrhizal symbiosis. A In control/ AM fungi uninoculated plants, the inner cortical cells expressed GFP-MtbHLHm1 (in green) in the nucleus and GFP-MtAMF1,3 in the plasma membrane (in yellow). There is a low signal GFP-MtbHLHm1 localized at the plasma membrane. In AM fungi inoculated plants, GFP- MtbHLHm1 (in green) was localized to the nucleus and to the membranes of cortical cells containing arbuscules. In the case of GFP-MtAMF1,3, it was localized at the PM of cortical cells and associated with membranes of cortical cells containing arbuscules (in yellow). **B** The presence of AM fungi/high Pi concentration trigger two forms of MtbHLHm1 in the AM fungi infected cortical cells: (a) one bound to the PM and the membranes of inner cortical cells containing arbuscules (or PAM) (in green/brown) and, (b) the free form (in green only) capable to bind to DNA in the nucleus to induce MtAMF1,3 transcription. Arbuscules are surrounded by the PAM, which is continuous with the plasma membrane of the root cell but is 4- to 10-fold larger in surface area [30]; thus, providing an extensive interface for nutrient exchange as well as low-affinity ammonium transport. To regulate low-affinity ammonium transport in such AM fungi infected cortical cells, i.e., *MtAMF1,3* transcription and its protein synthesis, both forms would be present. The membrane bound-MtbHLHm1 would be incapable of entering the nucleus to induce *MtAMF1,3* transcription (downregulation) while the free form would induce *MtAMF1,3* transcription (upregulation). The ratio of membrane bound-MtbHLHm1 and free MtbHLHm1 in AM fungi infected cortical cells could help in regulating low-affinity ammonium transport. C, cytoplasm; N, nucleus; PM, plasma membrane; PAM, the periarbuscular membrane; PCW, plant cell wall.

Supplementary Table S1. PCR Primers used in RT-PCR gene expression analysis.
For the amplification of target mRNA, PCR primers were designed for common *bHLHm1*, *AMT* and *AMF* genes present in Phytozome (V12)

Gene	Forward Primer 5′-3′	Reverse Primer 5′-3′
UBQ10	CCCTTCATCTTGTCCTTCGTCTG	CACCTCCAATGTAATGGTCTTTCC
bHLHm1	CCTACCGGAAATGAAAGACCG	GTGTATCTGATGTTGGAGGAC
AMF1;1	CAATGCCCCAGCTTCTCAGC	CGCCAACCAAAATGACTTCCAACC
AMF1;3	ATCGTGGGACTTGCACTGATGG	GGACATCCTTTGCATTTCA
AMF2	TCGGTGTAGGTGAGGCTTCCTTCA	AGCAGTTCCGGCTGGTATACACAT[illegible]
AMT1;1	AGATGAACTTGCGGGTATGG	TTGGAGTAGGAGTGGAAGAAGAA
AMT1;2	GCTTAGGTCTTCACCCGAGG	TGGCATGTTCTTCAGCCTCT
AMT1;3	TAAGGAAGTTTATGGCGGTTCTCC	ATCCAGACATCTCTTCCTCATG
AMT1;4	GGCGTTTGGTATCTGCTGGA	AACCTGCAATGCTCCAAACTTC
AMT2;1	TGGGGTAAAGGTGCACCGGCTT	AAACAAGTGAAGCCATAGGA
AMT2;2	TTGGGGTAAAGCTGGCCCTG	AGTCTCCAAACTACCGTCA
AMT2;3	CTACTTTCTAAGGCATCAAC	GAAATTCATTCTCCCAAGCAAAG
AMT2;4	CTTAGGGAAACCAAATGTAGCA	ATCAAAGTAAGACCAGCGA

Supplementary Table S2. Accession numbers of sequences used in this work

Protein Name (Symbol)	Accession (Phytozome12)	Accession (NCBI)
Ubiquitin 10 (UBQ10)	Medtr4g124610.1	ABD32351.2
basic Helix-Loop-Helix membrane 1 (bHLHm1)	Medtr2g010450.1	XP_003593344.2
Ammonium Facilitator 1;1 (AMF1;1)	Medtr2g010370.1	XP_003593336.2
Ammonium Facilitator 1;3 (AMF1;3)	Medtr4g092770.1	XP_003608336.2
Ammonium Facilitator 2 (AMF2)	Medtr5g030580.1	XP_003612923.1
Ammonium Transporter 1;1 (AMT1;1)	Medtr1g045550.1	XP_003590177.1
Ammonium Transporter 1;2 (AMT1;2)	Medtr7g113340.1	XP_003626273.1
Ammonium Transporter 1;3 (AMT1;3)	Medtr7g098930.1	XP_003625418.1
Ammonium Transporter 1;4 (AMT1;4)	Medtr1g079760.1	XP_003590923.2
Ammonium Transporter 2;1 (AMT2;1)	Medtr7g069640.1	XP_003623328.1
Ammonium Transporter 2;2 (AMT2;2)	Medtr8g095040.1	XP_003630386.1
Ammonium Transporter 2;3 (AMT2;3)	Medtr8g074750.1	XP_003629223.2
Ammonium Transporter 2;4 (AMT2;4)	Medtr7g115050.1	XP_003626447.1
